# Supplementary figures and images for: Tissue-specific transcriptional profiling of plasmacytoid dendritic cells reveals a hyperactivated state in chronic SIV infection
Source: PLoS Pathog. 2021 Jun 28;17(6):e1009674. doi: 10.1371/journal.ppat.1009674 (PMC8270445; doi:10.1371/journal.ppat.1009674)

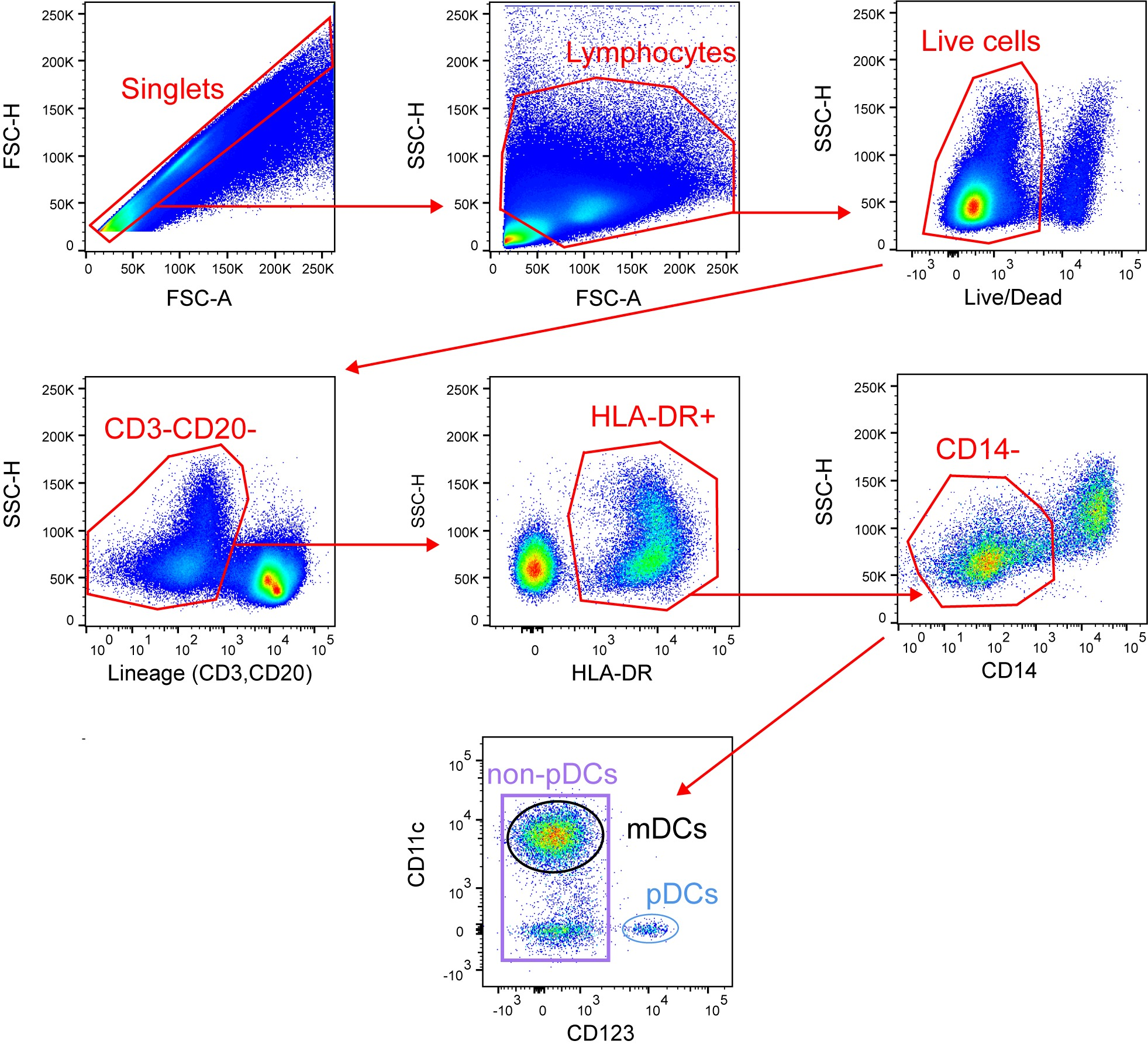

Supplement: S1 Fig — pDCs are defined as live CD3- CD20- HLA-DR+ CD14- CD11c- CD123+ leukocytes and shown with the blue circle in the final scatter plot. mDCs are defined as live CD3- CD20- HLA-DR+ CD14- CD11c+ CD123- leukocytes and shown with the black circle in the final scatter plot. Non-pDCs used in the ELISpot assay were sorted using the same strategy and are indicated by the purple gate (CD3- CD20- HLA-DR+ CD14- CD123-). (TIF) [file ppat.1009674.s001.tif]

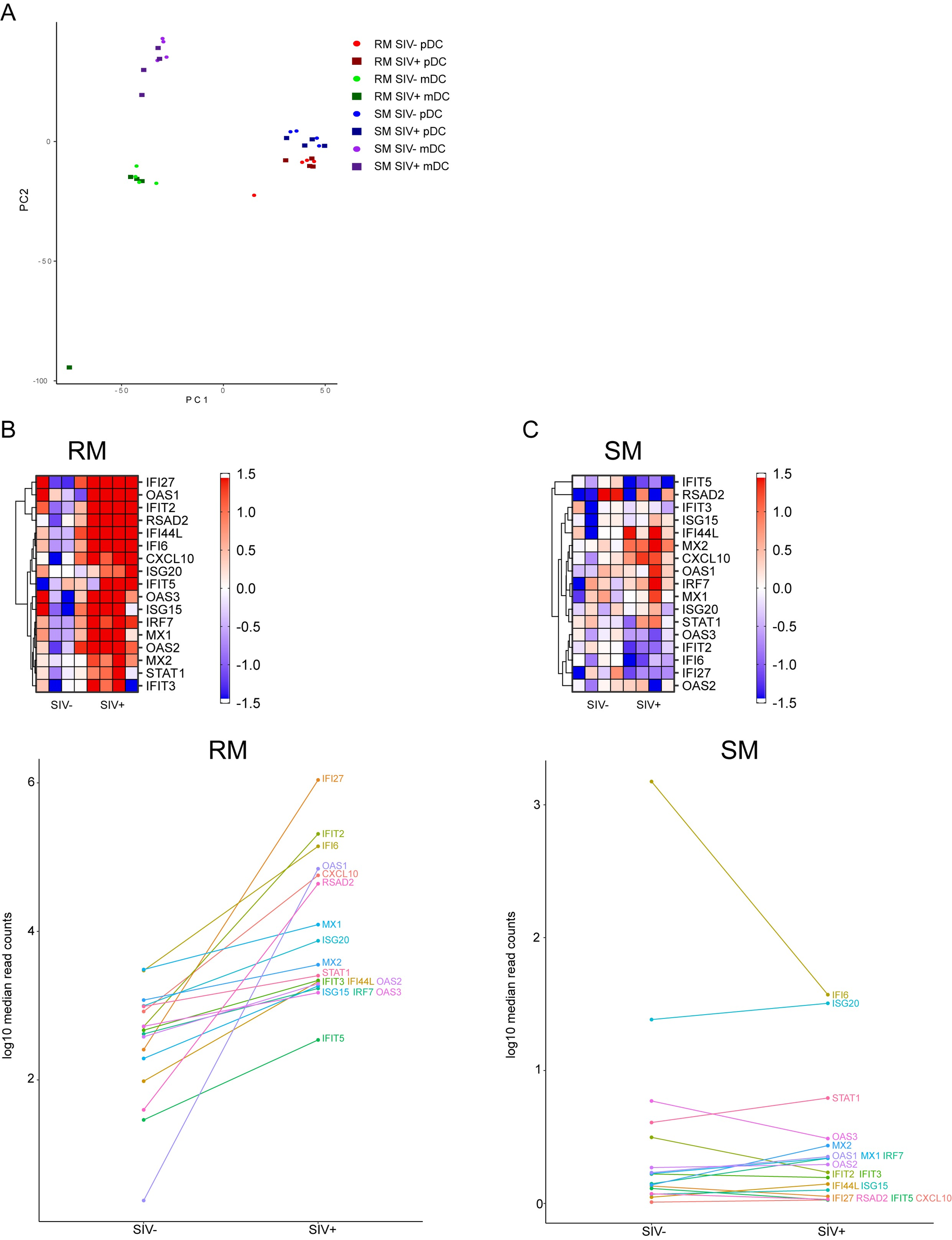

Supplement: S2 Fig — (A) Principal component analyses (PCA) of the transcriptomes of uninfected (circles) and infected (squares) pDCs and mDCs from RMs and SMs. RM pDCs are in red, RM mDCs are in green, SM pDCs are in blue and SM mDCs are in purple. Heat maps of 17 genes from an ISG signature panel representative of Type I IFN signaling for uninfected and SIV-infected RMs (B) and uninfected and SIV-infected SMs (C). The values shown were first transformed using the “rlog” method from DESeq2, data were normalized by subtraction of the median (on a per-gene basis) of the baseline samples (i.e. SIV- RM and SIV-SM) from each datapoint; RMs data was normalized by the SIV-RM median and SM data by the SIV-SM median. The color scale of the heatmap is set to maximal at fold-changes of -1.5 and 1.5. Normalized read counts (log10) for the 17 ISGs is shown in the bottom graphs. Dots show median normalized read counts (log10) of n = 4 for each experimental group. (TIF) [file ppat.1009674.s002.tif]

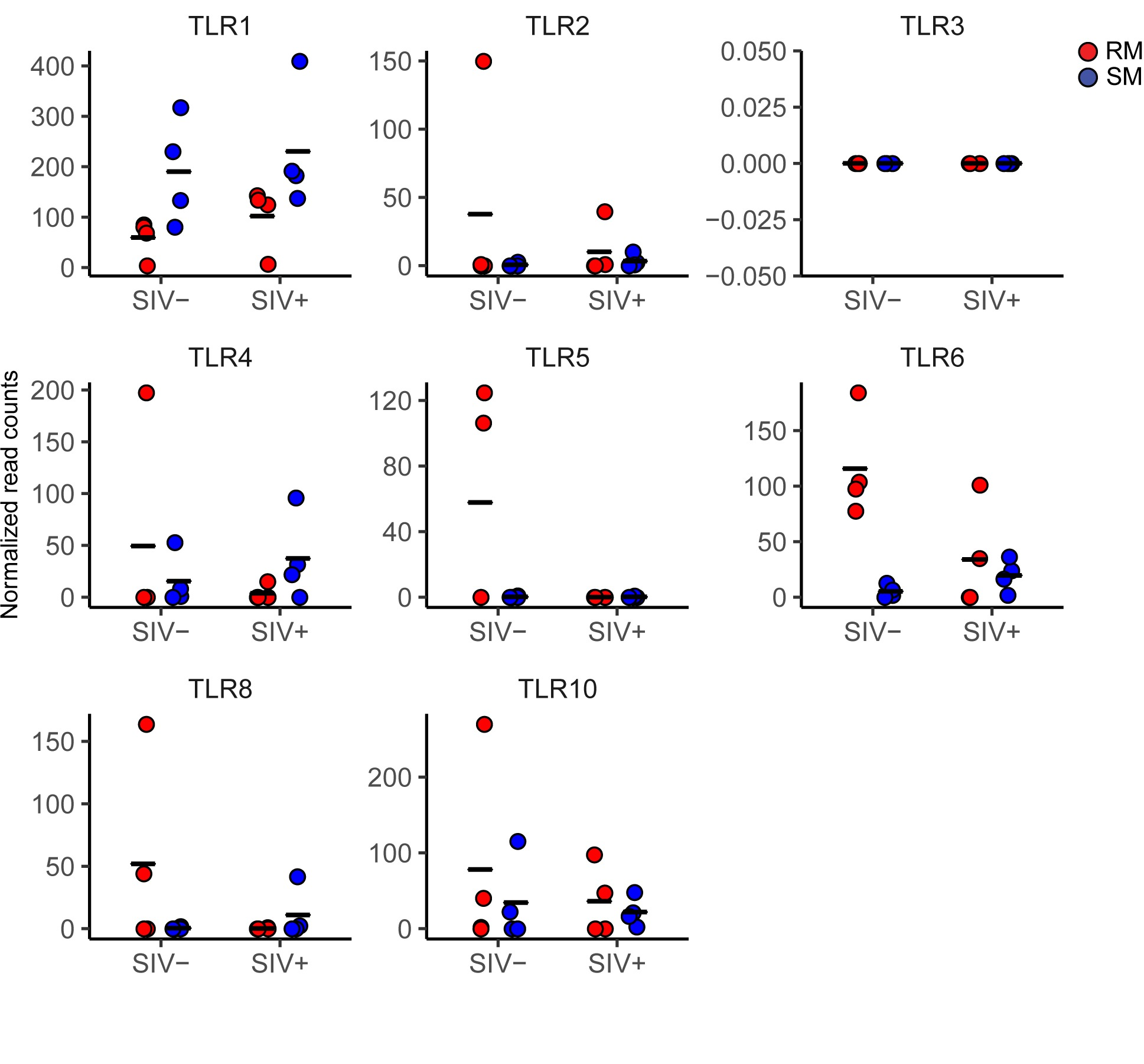

Supplement: S3 Fig — Normalised read counts for the cell surface (TLR1, 2, 4, 5, 6, 10) and intracellular (TLR3, 8) pattern recognition receptors in pDCs. All plots show values for individual RMs and SMs and black line indicates mean, n = 4 for each condition. (TIF) [file ppat.1009674.s003.tif]

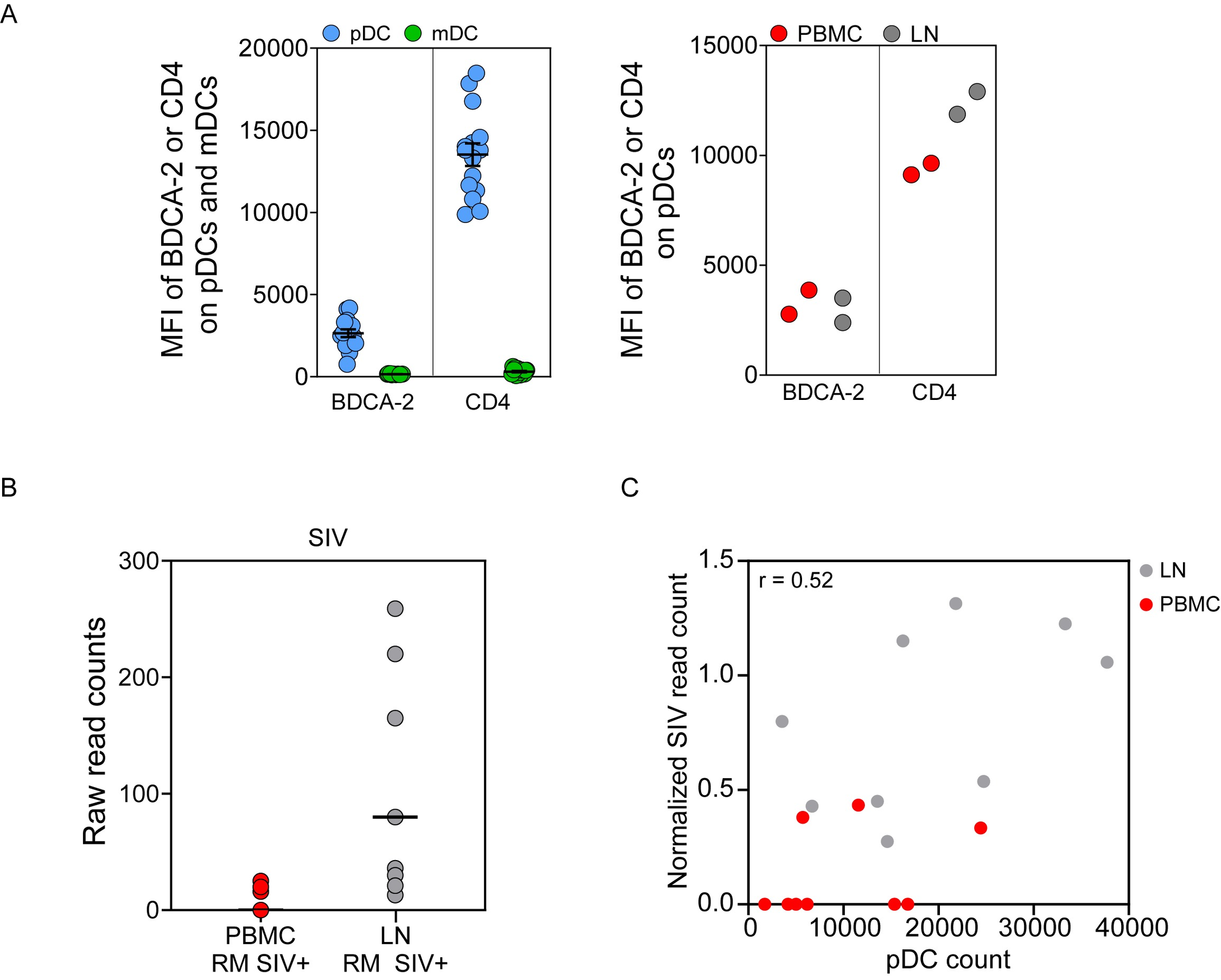

Supplement: S4 Fig — (A) Median fluorescence intensity (MFI) of CLEC4C/BDCA-2 or CD4 expressed on the surface of PB pDCs (blue circles) and mDCs (green circles) from 15 uninfected RMs (left) detected via flow cytometry. Median fluorescence intensity (MFI) of CLEC4C/BDCA-2 or CD4 expressed on the surface of PB (red) or LN (gray) pDCs from two uninfected RMs (right) detected via flow cytometry. Levels of CLEC4C/BDCA-2 or CD4 were measured on the CD11c- CD123+ or CD11c+ CD123- populations gated as shown in S1 Fig. (B) Raw read counts of SIV transcripts mapped to SIVmac239 in pDCs isolated from the blood (red) or lymph node (grey) of matched and unmatched SIV+ RM (left panel). Read counts are shown for individual RMs and the black bar indicates mean. Relationship between input pDC cell number and normalized SIV read count (right panel). (TIF) [file ppat.1009674.s004.tif]

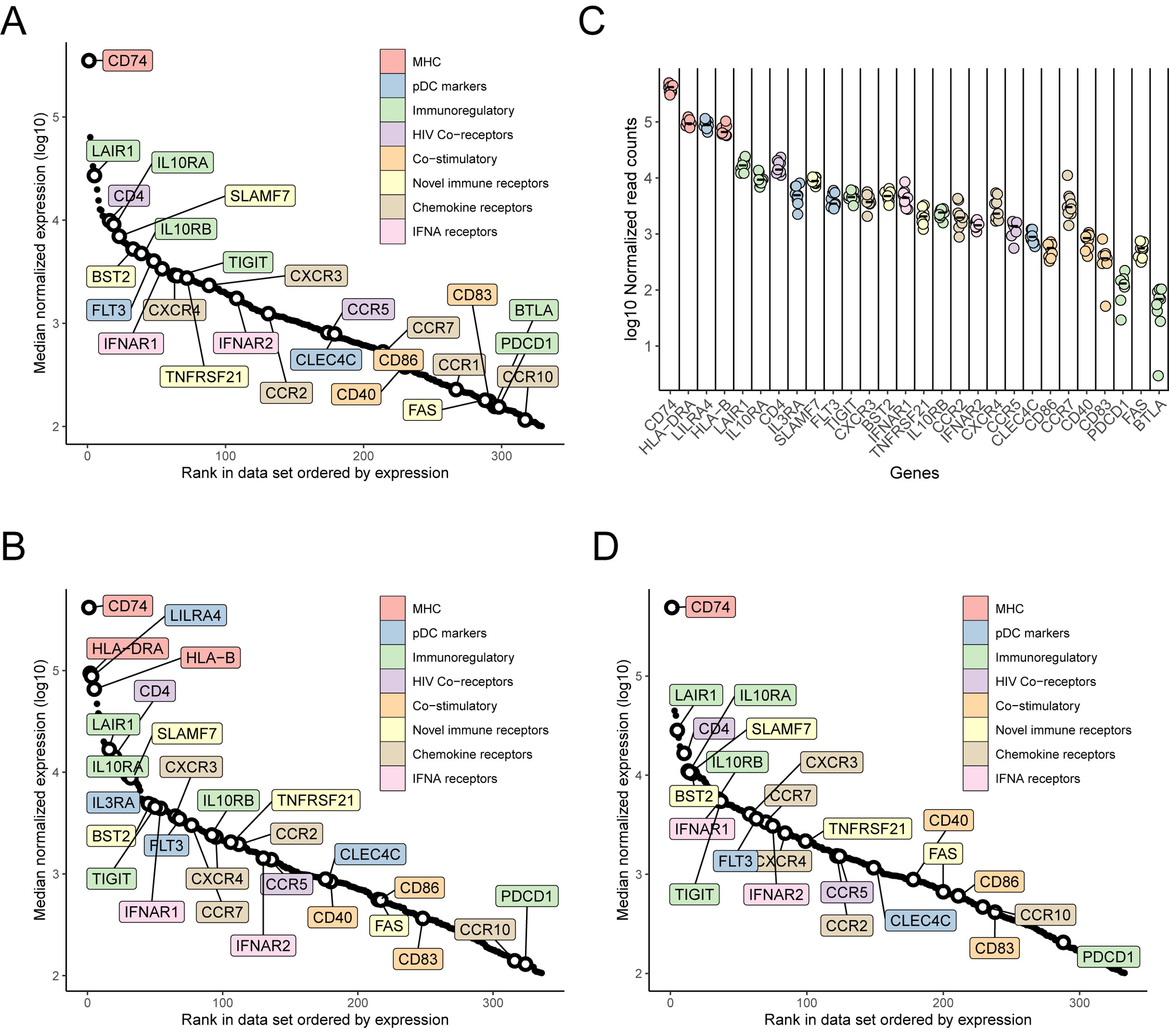

Supplement: S5 Fig — A. Expression (log10) of surface protein genes with the highest expression (median expression across all samples >100) in SIV+ RM PBMC samples (n = 9). Preliminary list of surface proteins obtained from http://wlab.ethz.ch/cspa/ with five additional genes of interest included (CLEC4C, CCR5, CCR2, CXCR4). Labelled genes of interest are color-coded according to their annotated immune function. B-D. The equivalent surface protein gene expression plots for LN-derived pDCs, B and C were aligned to GRCh38, D was aligned to MacaM. (TIF) [file ppat.1009674.s005.tif]

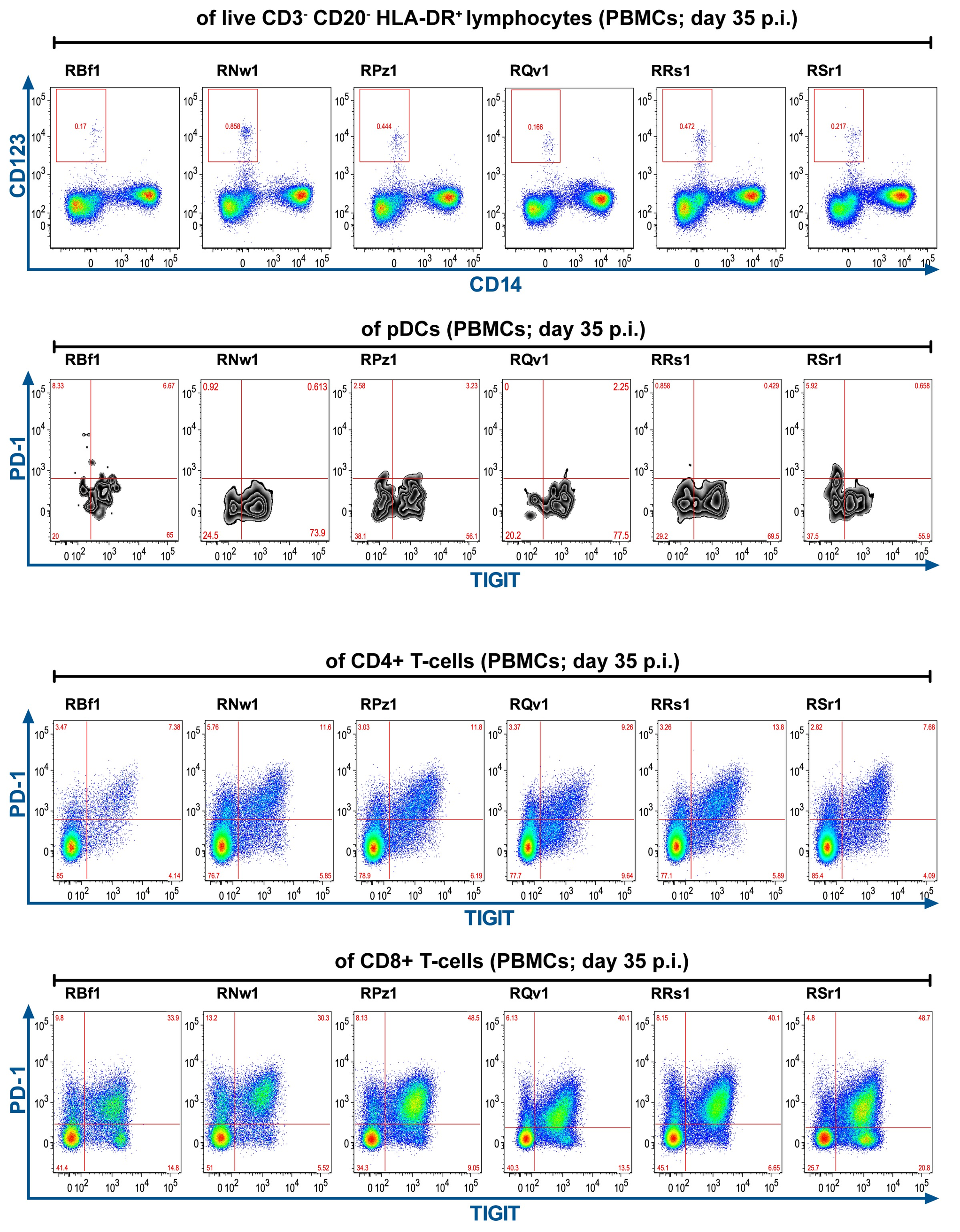

Supplement: S6 Fig — pDCs gated as CD14- CD123+ cells from live CD3- CD20- HLA-DR+ lymphocytes (top panel). Flow cytometry plots showing expression of PD-1 and TIGIT on peripheral blood pDCs, CD4+ or CD8+ T cells of six SIV-infected RMs at 35 days post-infection (bottom 3 panels). (TIF) [file ppat.1009674.s006.tif]

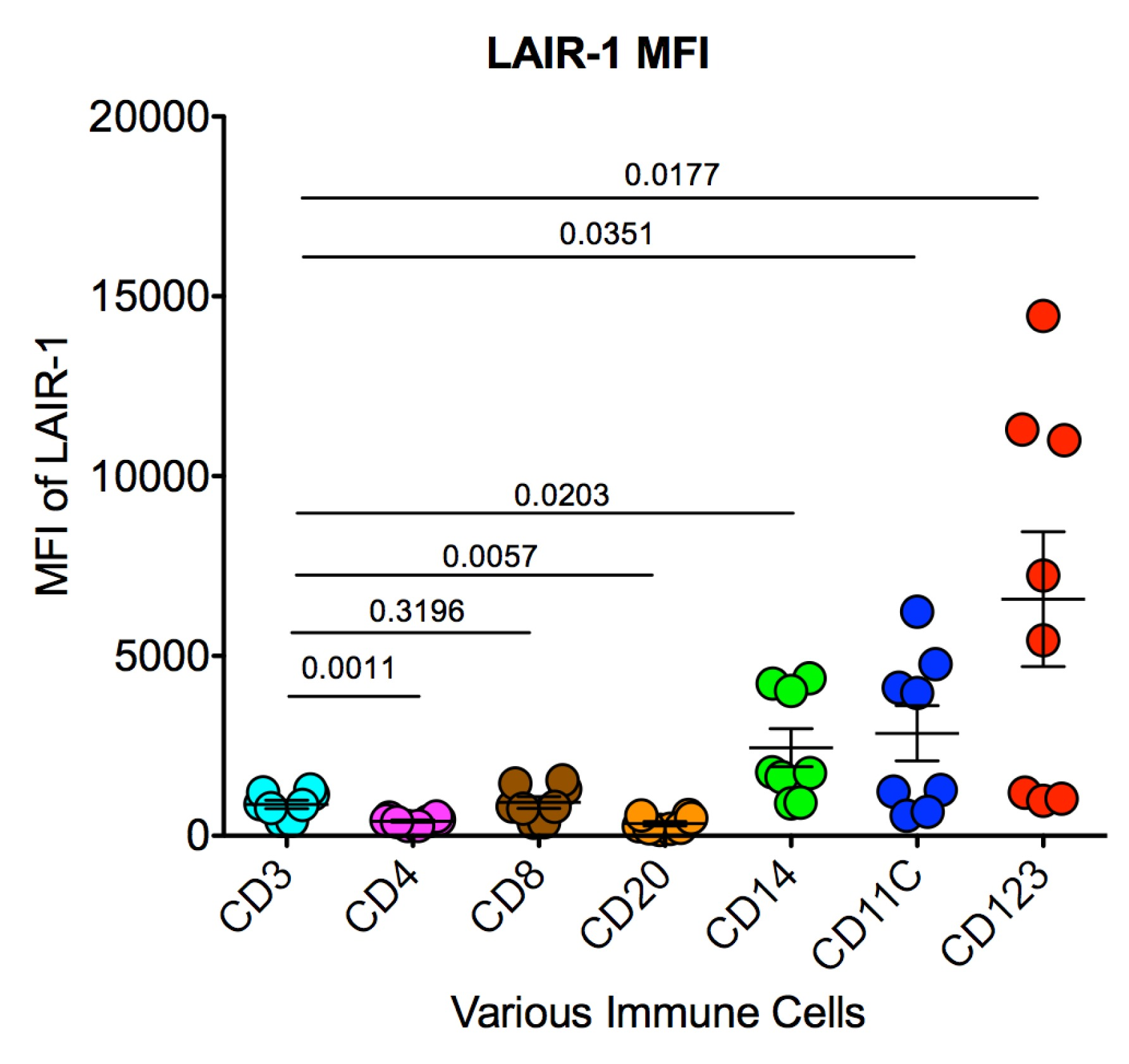

Supplement: S7 Fig — Median Fluorescence Intensity (MFI) of LAIR1 on total T cells (CD3+), CD4+ T cells, CD8+ T cells, B cells (CD20+), monocytes (CD14+), conventional dendritic cells (CD11c+) and plasmacytoid dendritic cells (CD123+) from eight SHIV-infected RMs at 16 weeks post-infection. Statistical comparisons were made using a paired T test. (TIF) [file ppat.1009674.s007.tif]

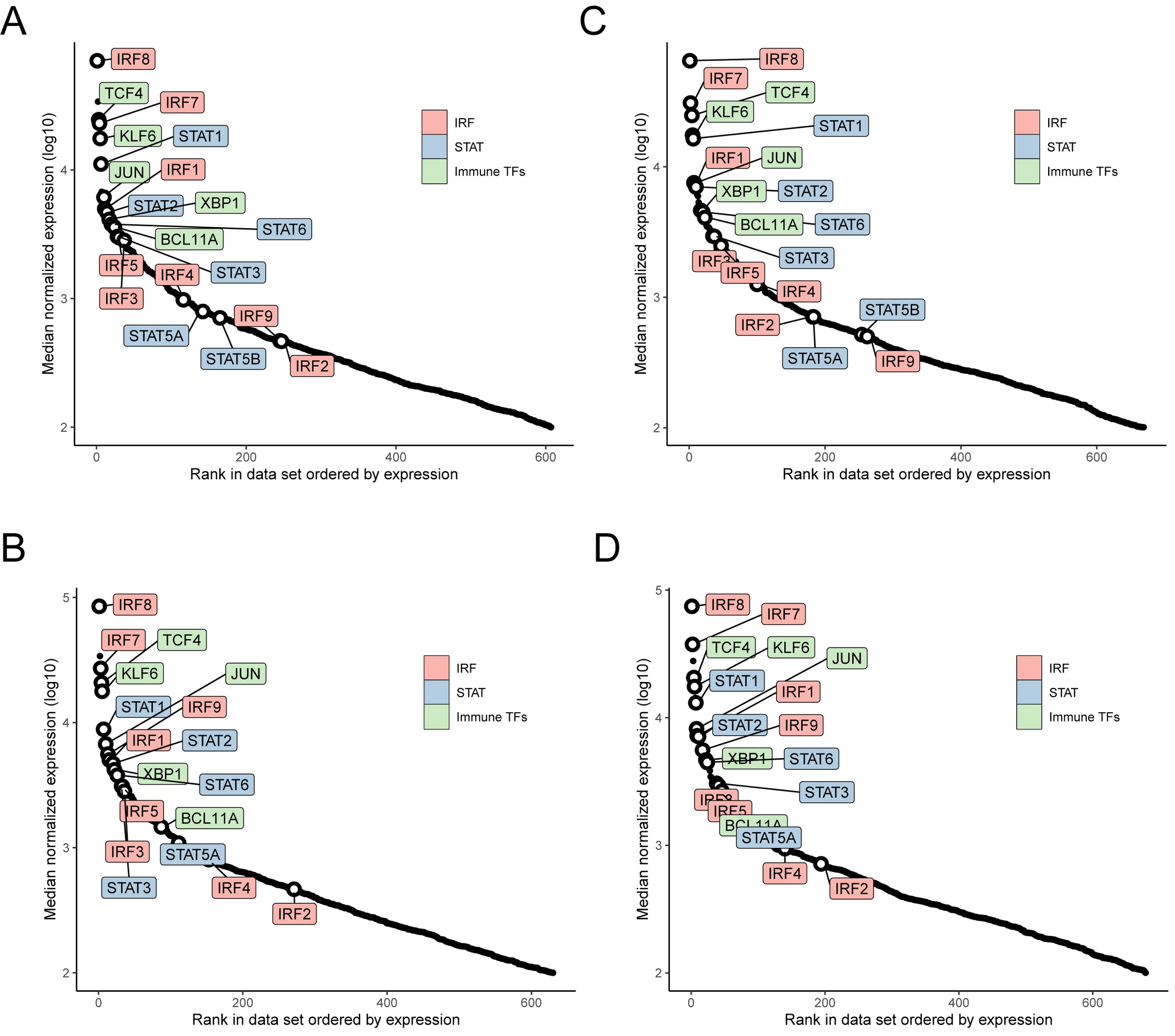

Supplement: S8 Fig — Expression (log10) of transcription factor genes with the highest expression (median expression across all samples >100) in SIV+ RM PB pDC samples (n = 9). Labelled genes of interest are color-coded according to their annotated functional families. Alignments to both GRCh38 (A) and MacaM (B) are shown. The equivalent plots for SIV+ RM LN-derived pDC samples (n = 9), with alignment to GRCh38 (C) and MacaM (B). (TIF) [file ppat.1009674.s008.tif]
