# Supplementary material for: Tissue-specific transcriptional profiling of plasmacytoid dendritic cells reveals a hyperactivated state in chronic SIV infection
Source: PLoS Pathog. 2021 Jun 28;17(6):e1009674. doi: 10.1371/journal.ppat.1009674 (PMC8270445; doi:10.1371/journal.ppat.1009674)
Supplement: Attachment — Submitted filename: Lee_et_al_Response_to_Reviewers.docx [file ppat.1009674.s013.docx]

***Rebuttal Letter:***

*Reviewer #1: In their manuscript, Lee and colleagues perform a cross-NHP species transcriptomic analysis of pDCs to evaluate their role(s) in ongoing HIV-induced immune activation. The investigator’s conclude in the pathogenic rhesus species, pDCs develop a hyperactivated state in lymph nodes during SIV infection. The study is mostly well done and very well-written, but there are some experimental and interpretive concerns that should be addressed.*

*Reviewer #2: Lee et al studied pDC in SIV rhesus macaque infection to further understand their role. They find that pDC appear to be recruited to lymph nodes during infection and have an enhanced activation state as demonstrated by RNA-seq. The authors claim that the pDC are driving T cell activation and exhaustion, although this is not formally demonstrated in functional assays. Data are shown that confirm that pDC are major IFNa producers. The authors also suggest that pDCs themselves are exhausted. It is unclear to this reviewer whether they are indicating that pDC in blood are exhausted or those both in LN and blood. If the pDC in LN in blood are exhausted, then why is IFNa being produced. This needs to be clarified. The manuscript has used RNA-seq technology to further delineate the role of pDC in SIV (and thus test in HIV) infection. This methodology may allow easier investigation of such a difficult to study cell population ex vivo.*

We thank the reviewers for their kind assessments of the work. Please see our responses to specific comments below, in which we note the addition of several new pieces of data:

1. *Supplementary Figure 2. New RNA-Seq data showing the ISG profile in mDCs.*
2. *Figure 5 – new flow cytometry data on 15 additional RMs describing the longitudinal kinetics of TIGIT expression on pDCs before and after SIV infection.*
3. *Figure 4 - new analysis of RNA-Seq data describing the RNA expression of CCR7 on pDCs between blood and LN has been added to Panel B of Figure 4.*
4. *Addition of pVL measurements for all animals to Supplementary Table 1.*
5. *In Figure 3 – as requested by reviewers – we have added new data showing protein detection of IFNA in LN pDCs in chronic SIV infection using immunofluorescence. These data are added as Figure 3 Panel D*
6. *In Figure 3 – as requested by reviewers – we have added new data in which we – mapped SIV RNA readcounts in the pDC RNA-Seq data between LN and blood. We found that SIV RNA is significantly higher in LN pDCs. These new data have been added as Figure 3 Panel F and Supplementary Figure 4B and C.*

***Responses to Reviewer comments:***

*Major Revisions:*

*Reviewer #1: The SMs were clearly infected much longer than rhesus. How was this evaluated as a potential confounding factor? It may be difficult to make a direct comparison at all here.

A similar question applies for the fact that the SMs were likely much much older than the rhesus used. This could have a major impact on ISG and other gene expression and should be clarified for the infected and uninfected animals in both species. This may also require some additional analyses.*

**Response:**

Figure 5 from Bosinger et al. J Clin Invest. 2009;119(12):3556-3572. <https://doi.org/10.1172/JCI40115>.

Thank you for pointing out the aspect of the study. In prior work, we published that the ISG and the type I interferon pathway in whole blood RNA samples of SIV infected Sooty Mangabeys infected with SIVsmm dissipates at timepoints >30 days (Shown in Rebuttal Figure above). The samples in chronic infection (labeled “Chr”) were collected at 180-224 days post-infection for both groups of animals. The sooty mangabeys used in the Bosinger et al. 2009 paper were at 5-11 years of age, and experimentally infected with SIVsmm. A similar profile was reported by Jacquelin et al. J Clin Invest. 2009;119(12):3544-3555. <https://doi.org/10.1172/JCI40093> when comparing gene expression in peripheral blood CD4+ cells from Rhesus Macaques and African Green Monkeys in acute and chronic SIV infection. Thus – in prior work, we and others have established that the absence of ISGs during chronic SIV infection occurs in non-geriatric adult natural hosts, at least in the blood and CD4+ compartments. It is not possible to repeat the experiments from the current study to control for age and duration of infection between the two NHP models due to the moratorium on de novo SIV infections of captive sooty mangabeys. This important caveat has now been addressed directly in the manuscript (see lines 748-757).

“Our study contains some drawbacks. The median age of the SIV-infected SMs was 17 years compared to 4 years for the RMs and the duration of infection of SMs was 11 years compared to 7.5 months for the SMs. In our prior work, where younger SMs were infected for a shorter amount of time, we observed a similar ISG response in the periphery at 180 days post-infection [19], which matches the results from the pDCs and mDCs in the current study. Age-dependent changes in innate immunity have been described, however the lack of an ISG response in the chronically infected SMs in the current study agrees with the observed rapid shutdown of IFN post-infection in nonpathogenic NHP hosts of SIV as reported by us and others [19-23].”

*Reviewer #1: It is unclear to this reviewer, how it was determined these are tissue-resident pDCs in LN, rather than pDC that may have migrated there or are blood contaminating. This should be rephrased unless specific confirmation of tissue-residency markers (CD69, CD49s, etc) were used. This does not undermine the disparate profiles of LN vs. circulating pDC, but given recent changes in how tissue-resident populations are identified, wording should be careful.*

**Response:** We thank the reviewer for highlighting this issue. We have reviewed the manuscript and rephrased our wording wherever required to change the implication of “tissue residence” to “lymph node homing” or “migratory”. We did not see any difference in expression of tissue residency markers (CD69 and CD49) between the two compartments. CCR7 was expressed at higher levels in the LN-derived pDCs than circulating pDCs, whereas CD62L (Gene ID: SELL) was very highly expressed in both compartments. The data for CCR7 has been included in **panel B** of **Figure 4** and addressed in the text in lines 520-523:

“We observed that the read counts of CCR7 were significantly higher in the LN-derived pDCs compared to the PB pDCs, consistent with these cells homing to LNs (median read count, LN: 3181 vs PBMC: 562, 4.3-fold induction, padj = 0.0029) (Figure 4B).”

See the following lines for rephrasing of lymph node “resident” to “homing” or similar: 12, 33, 123, 133, 137, 449, 485, 566, 661, 670, 701, 703, 705.

*Reviewer #1: It would also be useful to confirm IFN production in the peripheral and LN pDCs by ICS or ELISPOT or ELISA to verify the conclusions by transcripts.*

**Response:** We have now included new data in which we performed immunofluorescence studies to demonstrate that (i) IFNα protein is detectable in LNs obtained from RMs during chronic SIV infection, and (ii) the IFNα protein was exclusively found co-localized in cells expressing CD303/BDCA2, a unique marker for primate pDCs. We feel that these new data demonstrating IFNα at the protein level, provide strong validation of our RNA-Seq observations.

These new data are the first, in our knowledge, to show IFNa protein in pDCs during chronic SIV infection. However, it should be noted that the production of IFN I in LNs from pDCs (or cells with a pDC morphology) has been shown in RMs, AGMs and SMs in *acute SIV infection* 14 days post infection (Harris et al. J Virol. 2010; 84(15):7886-7891. doi:10.1128/JVI.02612-09). And in human/HIV studies, Lehmann and colleagues used ICS to show high levels of IFN-α production by unstimulated, lymph node homed pDCs in chronically infected, treatment-naïve HIV-1 positive individuals (Lehmann et al. PLoS One. 2010; 5(6):e11110. doi:10.1371/journal.pone.0011110).

The data is included in **panel D** of Figure 3 and addressed in text in lines 477-483.

“To confirm the production of IFN-α in LN-derived pDCs at the protein level, we performed multiplex staining of LN samples from another cohort of three chronically infected RMs for CD303/BDCA-2 and IFN-α with fluorescently labelled antibodies. We observed that the majority of CD303+ cells were localized in the extra-follicular, medullary cord regions of the LN and that there was a strong co-localization of CD303+ and IFN-α cells (Figure 3D). These data demonstrate that in chronic untreated SIV infection, there is production of IFN-α protein from pDCs in the LN.”

*Reviewer #1: Were ISGs in non-pDCs upregulated correlating within pDCs? Since the pDC may be a primary source this would seem a natural analysis rather than necessarily looking at ISGs in the pDCs themselves.*

**Response:** To address this we have added new experimental data, in which we sorted myeloid DCs (mDCs) (CD3- CD20- HLA-DR+ CD14-CD11c+ CD123-) from the same RM and SM PBMC samples shown in Figure 2 and performed RNA-Seq in the same batch as the original pDC data. These sorts were done simultaneously with the original pDC sorts. The ISG profile from SM and RM mDC has now been included as **Supplementary figure 2** in the manuscript. These data demonstrate very clearly that ISG signatures are strongly upregulated in mDCs from infected RMs, but are not detectable at elevated levels in mDCs from SMs. We have added the following lines (392-396) to the manuscript.

“Since pDCs are the primary source of IFN I in response to SIV, we determined the ISG response profile in non-pDCs (myeloid dendritic cells, mDCs (defined as (CD3- CD20- HLA-DR+ CD14-CD11c+ CD123-), gating shown in **Supplementary Figure S1**) that were sorted from the same animals in parallel with pDCs. Consistent with the data from pDCs, a strong upregulation in all ISGs in our panel was seen in mDCs derived from SIV-infected RMs, but not SMs (**Supplementary Figure S2**).”

*Reviewer #1: The authors make the argument that hyperactivated pDCs may have a role in lentivirus disease. Were no virologic measurements performed? Particularly in LN.*

**Response:** We thank the reviewer for pointing out this omission. We have now included plasma VLs for all animals in Supplementary Table 1. Of note – whereas the pVLs for the RMs were taken within ~1 week of the date of the flow sorting, pVLs for the SMs were assessed 1 yr prior to sampling for 2 animals, and 3 yrs prior for the other 2 animals. While this is not ideal, we have demonstrated previously that pVLs for Sooty Mangabeys remain stable over time during chronic infection. Viral loads of 78 SM were followed over 5 years in previous study of cohort at Yerkes. Taaffe et al. J Virol. 2010; 84(11):5476-5484. Doi: 10.1128/JVI.00039-10. We were not able to obtain LN-associated VLs at the time of flow sorting due to sampling limitations – we used all available material for obtaining pDCs.

We have added the following lines to the results:

“To profile the transcriptional response in pDCs … (9-12 years infected, **median plasma viral load (PVL) of 2.58x104 copies/mL).** To directly contrast these data with transcriptional profiles from pDCs in pathogenic SIV infection … (duration 179-237 days, **median PVL of 2.39x105 copies/mL).** … These animals were sampled at 97-370 days post-infection with a **median PVL of 3.16x105 copies/mL.”**

See lines 346, 348 and 353 in the manuscript

*Reviewer #2: No functional data is shown that pDC are indeed exhausted or whether they drive exhaustion of other cell types.*

**Response:** We thank the reviewer for this observation, and carefully considered how to address this question. While we believe that a co-culture system of lymphocytes with in vitro activated pDCs would likely yield increased expression of exhaustion related molecules due to Type I IFN, we felt that these would have limited value due to the supraphysiologic nature of in vitro simulation. While outside of the scope of this manuscript, we wish to note that we are currently investigating the ability of pDCs to drive exhaustion in T cells in vivo during SIV infection in a large study comprised of 15 RMs (R01 AI136990). However, the reviewer makes an important point, and we have now provided caveats to our observations in the Discussion (see lines 730-733).

“Our observation of T cell exhaustion pathways enriched in pDCs are consistent with a model in which hyperactive pDCs localize in T cell areas of LNs and contribute to T cell dysfunction and exhaustion, although this needs to be tested by functional studies in vivo.”

*Minor Revisions:*

*Reviewer #2: Clarify if the macaques in figure one that are SIV infected are on cART or not.*

**Response:** This has been rectified in both the main text and figure legend (see lines 358-359)
 *-Please add the p value for the uninfected counterparts on line 347, and in line 359*

**Response:** This has been rectified in the text (see line 388)

*-Fig 3d, please show error bars.*

**Response:** Error bars have been added to figure 3d, showing SD.

*-Also In fig 3 Lee et al shows shows constitutive expression in infected animals of RNA, is this true for protein expression?, this condition could be added in fig 3d, ie add pdc from uninfected animals at the same concentration without stimulation.*

**Response:** We have addressed this by use of multiplex immunofluorescence experiments demonstrating IFN-α protein detected in pDCs in LNs of chronically infected RMs (see major revisions section for comments from reviewer #1).

*-Do we know whether the pDC in LN versus PB or both contain SIV virus as others have shown? If so, can this explain some of the findings?*

**Response:** To address this, we have quantitated the RNA-Seq reads mapping to the SIVmac239 genome in the LN- and PBMC-derived pDCS. We found that LN-derived pDC had significantly higher levels of SIV RNA (by RNA-Seq based detection) than the PBMC-derived pDC. We have added these new SIV RNA data in **Figure 3F**, and as **Supplementary Figure 4B, C**.

In several older studies, groups have investigated whether or not pDCs can support HIV/SIV replication. The clearest in vitro data has been provided by Koup and colleagues (Loré et al. J Exp Med (2005) 201 (12): 2023–2033. https://doi.org/10.1084/jem.20042413) who demonstrated that pDCs could support a moderate level of HIV replication when infected in vitro, and that pDC exposed to HIV in vitro could transfer virus to enhance CD4+ T cell infection. The question of whether pDCs contain relevant HIV/SIV transcripts in vivo, was perhaps most clearly addressed by Patterson and colleagues (Donagy et al. Blood. 101:4505–4511. https://doi.org/10.1182/blood-2002-10-3189) They showed that pDCs from blood of ART-naïve PLWH did not have evidence of HIV provirus in peripheral blood samples. We are unaware of any prior studies comparing matched LN and PBMC HIV/SIV RNA levels in pDCs.

Given our new data – we concur with the reviewer – that the differential levels of SIV RNA associated with pDCs from PBMCs vs LN may explain the dichotomy in their IFNA production. We have added a sentence in both the Results and Discussion describing these data, and proffering it as a possible explanation for the increased IFNA, but given that a formal demonstration of this would take additional experiments, did not expand on this interpretation.

We have added the following text to the manuscript (lines 497-503):

“Lastly, we investigated if the level of cell-associated SIV RNA was different between LN and PB pDCs by quantitating the normalized RNA-Seq reads mapping to a genomic reference of SIVmac239. As shown in Figure 3F, we observed that samples from LN harboured a significantly higher number of reads mapping to SIV (median log10 normalized read counts LN: 0.799 vs PBMC: 0, p = 0.0005). This observation was consistent when quantifying raw, non-normalized SIV read counts (Supplementary Figure 4B), and SIV read count was independent of the input number of pDCs (Supplementary Figure 4C).”

and lines 718-725 in the discussion

“Interestingly, we also observed that pDCs from LNs had significantly elevated levels of cell-associated SIV RNA transcripts detected by RNA-Seq. Work by Koup and colleagues demonstrated that pDCs could support a moderate level of HIV replication when infected in vitro [97], however, in vivo, others have failed to detect HIV provirus in pDCs from peripheral blood of ART-naïve PLWH [98]. Our observations of cell-associated SIV transcripts, cannot address if pDCs harbour SIV replication in tissues. However, the increased activation profiles of the LN pDCs may be partially due to being exposed to higher levels of SIV in the LN, but additional work will be required to test this formally.”

*-Is it confirmed that pDC in one compartment are CCR7 surface positive in one compartment over another?*

**Response:** In humans, HIV infection is associated with higher CCR7 expression on circulating pDCs and increased numbers in LNs (Lehmann et al. PLoS One. 2010; 5(6):e11110. doi:10.1371/journal.pone.0011110). In vitro exposure of pDCs to HIV (Fonteneau et al. J Virol. 2004; 78(10):5223-5232. doi: 10.1128/jvi.78.10.5223-5232.2004) or TLR7 ligands (Tavano & Boasso. PLoS One. 2014; 9(2):e89414. https://doi.org/10.1371/journal.pone.0089414) drastically increase their surface expression of CCR7 and migratory capacity to CCR7 ligands. In macaques, the frequency of pDCs in the blood expressing CCR7 has been reported to significantly increase after SIV infection (Kwa et al. Blood. 2011; 118(10): 2763-2773. doi: 10.1182/blood-2011-02-339515) and correlate with higher accumulation in LNs (Mandl et al. Nat Med. 2008; 14(10): 1077-1087. doi: 10.1038/nm.1871); an enrichment of CCR7^high^ pDC in LNs during acute SIV infection has also been reported in cynomolgus macaques (Bruel et al. PLoS Pathogens. 2014; 10(1): e1003915. https://doi.org/10.1371/journal.ppat.1003915). However, we were unable to find a direct comparison of CCR7 levels on pDCs between LN and blood in the literature.

We examined our RNA-Seq data and observed that, indeed, LN pDCs had significantly elevated levels of CCR7 mRNA expression relative to those from blood (median readcount, LN: 3181 vs PBMC: 562, 4.3-fold induction, padj = 0.0029).

We have included these new CCR7 data into the manuscript as an additional graph in **figure 4B** with the following text in lines 520-523:

“We observed that the read counts of CCR7 were significantly higher in the LN-derived pDCs compared to the PB pDCs, consistent with these cells homing to LNs (median read count, LN: 3181 vs PBMC: 562, 4.3-fold induction, padj = 0.0029) (Figure 4B).”

*-TIM-3 has been associated with pdC exhaustion in mice and humans, what was TIM-3 levels in these pDCs.*

**Response:** Recent work has demonstrated that TIM-3 is expressed on human pDCs, is elevated on pDCs in HIV-infected persons, and its expression was associated with attenuated production of IFNα and TNFα (Schwartz et al. J Immunol. 2017; 198(8): 3181-3194. doi: 10.4049/jimmunol.1601298), and its surface level has been reported to be predictive of ART-driven reduction of PVL (Font-Haro et al. Viruses. 2018; 10(4): 154. https://doi.org/10.3390/v10040154). TIM-3 has also been reported on pDCs in mouse models of cancer (Chiba et al. Nat Immunol. 2012; 13: 832-842. https://doi.org/10.1038/ni.2376) suggesting that its expression on pDCs is conserved in mammals. However, detection of TIM-3 on rhesus macaque cells has remained a technical challenge: neither our laboratory, nor collaborators, have had success with any of the anti-human TIM-3 clones currently available for reproducibly detecting rhesus TIM-3. In the current dataset, we have examined the RNA expression of TIM3/HAVCR2 and found it to be extremely low, in most cases the median expression was ~5 readcounts.

We have amended the manuscript by including discussion of TIM3 and noting the detection of negligible levels of transcripts of this exhaustion marker in PB and LN pDCs from SIV-infected RMs, see lines 613-615.

“In contrast, the expression of TIM3, an exhaustion marker that has been shown to be highly expressed on HIV-1 infected human pDCs [85, 86], was negligible in PB and LN pDCs from SIV-infected RMs.”

*-Exhaustion markers TIGIT is elevated in pdC from SIV infected animals but this reviewer does not see expression of pDC from uninfected animals. In fig 5c, it is unclear whether the pDC are from periphery or LN.*

**Response:** We have now included new data in the manuscript from 15 animals before and after SIV infection (at 42 days post-infection – chronic), showing levels of TIGIT expression on peripheral blood pDCs. This has been addressed in lines 629-633. In addition, we kept the original data in Figure 5D showing TIGIT vs PD1, have added text to the Figure 5 legend clarifying that these samples were from peripheral blood.

“To investigate the kinetics of surface expression of TIGIT on PB pDCs before and after SIV infection, we performed flow cytometry on an independent set of 15 RMs. We observed a high frequency of TIGIT+ pDCs prior to infection that was maintained in early chronic infection (Day 42) (Figure 5E, representative staining of 3 RMs and Figure 5F).”

FORMATTING

Figure Files:

While revising your submission, please upload your figure files to the Preflight Analysis and Conversion Engine (PACE) digital diagnostic tool, [https://pacev2.apexcovantage.com](https://pacev2.apexcovantage.com/). PACE helps ensure that figures meet PLOS requirements. To use PACE, you must first register as a user. Then, login and navigate to the UPLOAD tab, where you will find detailed instructions on how to use the tool. If you encounter any issues or have any questions when using PACE, please email us at [figures@plos.org](mailto:figures@plos.org).

Data Requirements:

Please note that, as a condition of publication, PLOS' data policy requires that you make available all data used to draw the conclusions outlined in your manuscript. Data must be deposited in an appropriate repository, included within the body of the manuscript, or uploaded as supporting information. This includes all numerical values that were used to generate graphs, histograms etc.. For an example see here on PLOS Biology: <http://www.plosbiology.org/article/info%3Adoi%2F10.1371%2Fjournal.pbio.1001908#s5>.

Reproducibility:

To enhance the reproducibility of your results, PLOS recommends that you deposit laboratory protocols in protocols.io, where a protocol can be assigned its own identifier (DOI) such that it can be cited independently in the future. For instructions see [http://journals.plos.org/plospathogens/s/submission-guidelines#loc-materials-and-methods](http://journals.plos.org/plospathogens/s/submission-guidelines)

*In compliance with data protection regulations, you may request that we remove your personal registration details at any time.* [*(Remove my information/details)*](https://www.editorialmanager.com/ppathogens/login.asp?a=r)*. Please contact the publication office if you have any questions.*
